# A Computational Screen for Regulators of Oxidative Phosphorylation Implicates SLIRP in Mitochondrial RNA Homeostasis

**DOI:** 10.1371/journal.pgen.1000590

**Published:** 2009-08-14

**Authors:** Joshua M. Baughman, Roland Nilsson, Vishal M. Gohil, Daniel H. Arlow, Zareen Gauhar, Vamsi K. Mootha

**Affiliations:** 1 Center for Human Genetic Research, Massachusetts General Hospital, Boston, Massachusetts, United States of America; 2 Broad Institute of MIT and Harvard, Cambridge, Massachusetts, United States of America; 3 Department of Systems Biology, Harvard Medical School, Boston, Massachusetts, United States of America; University of Geneva Medical School, Switzerland

## Abstract

The human oxidative phosphorylation (OxPhos) system consists of approximately 90 proteins encoded by nuclear and mitochondrial genomes and serves as the primary cellular pathway for ATP biosynthesis. While the core protein machinery for OxPhos is well characterized, many of its assembly, maturation, and regulatory factors remain unknown. We exploited the tight transcriptional control of the genes encoding the core OxPhos machinery to identify novel regulators. We developed a computational procedure, which we call expression screening, which integrates information from thousands of microarray data sets in a principled manner to identify genes that are consistently co-expressed with a target pathway across biological contexts. We applied expression screening to predict dozens of novel regulators of OxPhos. For two candidate genes, *CHCHD2* and *SLIRP*, we show that silencing with RNAi results in destabilization of OxPhos complexes and a marked loss of OxPhos enzymatic activity. Moreover, we show that SLIRP plays an essential role in maintaining mitochondrial-localized mRNA transcripts that encode OxPhos protein subunits. Our findings provide a catalogue of potential novel OxPhos regulators that advance our understanding of the coordination between nuclear and mitochondrial genomes for the regulation of cellular energy metabolism.

## Author Summary

Respiratory chain disorders represent the largest class of inborn errors in metabolism affecting 1 in every 5,000 individuals. Biochemically, these disorders are characterized by a breakdown in the cellular process called oxidative phosphorylation (OxPhos), which is responsible for generating most of the cell's energy in the form of ATP. Sadly, for approximately 50% of patients diagnosed, we do not know the molecular cause behind these disorders. One possible reason for our limited diagnostic capability is that these patients harbor a mutation in a gene that is not known to act in the OxPhos pathway. We therefore designed a computational strategy called expression screening that integrates publicly available genome-wide gene expression data to predict new genes that may play a role in OxPhos biology. We identified several uncharacterized genes that were strongly predicted by our procedure to function in the OxPhos pathway and experimentally validated two genes, *SLIRP* and *CHCHD2*, as being essential for OxPhos function. These genes, as well as others predicted by expression screening to regulate OxPhos, represent a valuable resource for identifying the molecular underpinnings of respiratory chain disorders.

## Introduction

Mitochondrial oxidative phosphorylation (OxPhos) is central to energy homeostasis and human health by serving as the cell's primary generator of ATP. The core machinery underlying OxPhos consists of approximately 90 distinct protein subunits that form five complexes residing in the inner mitochondrial membrane. Complexes I through IV comprise the oxygen-dependent electron transport chain responsible for driving the generation of ATP by complex V. OxPhos is the only process in the mammalian cell under dual genetic control: thirteen essential structural subunits are encoded by mitochondrial DNA (mtDNA) while remaining subunits are encoded by nuclear genes, and are imported into mitochondria [1]. The biogenesis of OxPhos requires many accessory factors responsible for replicating mtDNA as well as transcribing and translating the mitochondrial mRNAs (mtRNA) [2],[3]. Furthermore, the mtDNA-encoded subunits must be coordinately assembled with the nuclear-encoded subunits and metal co-factors to form functional complexes, a process likely requiring far more assembly factors than are currently known [4]. Dysfunction in any of these processes or in the OxPhos machinery itself may result in a respiratory chain disorder, a large class of inborn errors of metabolism [5]. For approximately 50% of patients with respiratory chain disorders, the underlying genetic defect remains unknown, despite excluding obvious members of the OxPhos pathway [4], [6]–[8]. Many of these disorders are likely due to genetic defects in currently uncharacterized OxPhos assembly or regulatory factors.

The OxPhos structural subunits exhibit tight transcriptional regulation that offers a strategy for identifying its non-structural regulators based upon shared patterns of co-expression in microarray experiments [9],[10]. In fact, our laboratory used this approach to identify the gene *LRPPRC*, which encodes a critical regulator of mtRNA and when mutated is the underlying cause of a respiratory chain disorder called Leigh Syndrome French-Canadian variant [11]. However, while successful in identifying *LRPPRC*, this previous analysis used only one data set interrogating tissue-specific gene expression [12],[13]. Such co-expression analyses that rely upon individual contexts are not ideal for functional prediction because they are subject to inherent limitations of microarray experiments including technical artifacts, experimental bias and real but confounding correlations with functionally distinct pathways [14].

To overcome these limitations and to generalize our previous approach, we reasoned that large-scale integration across many independent microarray experiments, each surveying a different biological context, would help distinguish genuine co-regulation from random co-expression by identifying genes that consistently co-express with OxPhos. In the yeast *Saccharomyces cerevisiae*, several groups have performed such expression data integration studies to predict protein function [15]–[17]. With the recent availability of large repositories of mammalian microarray data, it is now possible to apply similar approaches to functionally classify uncharacterized human proteins [18],[19]. Studying mammalian data is especially important for OxPhos given that the mammalian OxPhos pathway differs significantly from the yeast counterpart. For example, *S. cerevisiae* lacks a proton pumping complex I, the largest OxPhos complex in human cells consisting of forty-five distinct protein subunits [20] and a common target of respiratory chain disease [21],[22]. Furthermore, mammalian mtDNA is circular, whereas yeast mtDNA can form linear concatemers [23]. Moreover, mammalian mtRNA processing differs markedly from *S. cerevisaie* as mammalian mtRNA does not contain introns and is polyadenylated [24].

In the present paper, we introduce a computational methodology, called “expression screening”, that takes advantage of the growing wealth of freely available mammalian microarray data to search for genes that exhibit consistent co-expression with a given “query” gene set. Applying this procedure to the mammalian OxPhos pathway revealed a number of putative regulators that now emerge as attractive candidate genes for OxPhos disorders. We experimentally validated two genes, *CHCHD2* (coiled-coil-helix-coiled-coil-helix domain containing 2) and *SLIRP* (SRA-stem loop interacting RNA-binding protein; also known as *C14orf156*) as essential for OxPhos function. We further characterized SLIRP as a RNA-binding domain containing protein necessary for the maintenance of mtRNA protein-encoding transcripts and whose robust co-expression with the nuclear OxPhos subunits provides a putative regulatory link between nuclear and mitochondrial gene expression.

## Results

**Figure 1:**
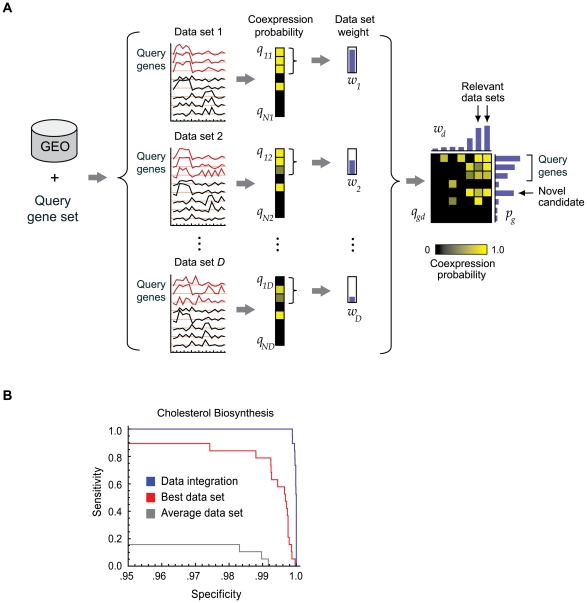
Overview and validation of expression screening. (A) Given a set of transcriptionally regulated genes as input (query gene set), expression screening interrogates a compendium of *D* microarray data sets (GEO) to produce a matrix of probabilities *q_gd_* of co-expression with the query genes for each of *N* genes (rows) measured in *D* data sets (columns). Each data set is assigned a weight, *w_d_* (vertical bars), according to the intra-correlation of the query gene set. A robust Bayesian data integration procedure is used to compute an integrated probability *p_g_* of co-expression for each gene (horizontal bars). (B) Cross-validated receiver-operator curve reporting the recovery of the query cholesterol biosynthesis gene set. Blue line: full expression screen data integration (1,427 data sets). Red line: the single best data set. Gray line: a data set with median weight.

We developed a computational procedure called “expression screening” (Figure 1), which applies large-scale co-expression analysis to a compendium of microarray experiments to predict genes with a functional role in a given pathway, such as OxPhos. We first assembled a compendium of microarray data sets by downloading publicly available expression data from the NCBI Gene Expression Omnibus [18]. We focused on mammalian biology by selecting human and mouse data, and avoided cross-platform discrepancies by limiting our analysis to data from Affymetrix oligonucleotide arrays. To ensure high quality data was used in downstream co-expression analyses, we removed small (n<6) data sets and duplicated experiments. Since previous studies have largely focused on tissue-specific gene expression [10],[11],[13] we decided to instead focus on datasets that measure changes in gene expression within individual tissues or cell types in response to various stimuli. We therefore excluded data sets containing multiple tissues. These filtering steps resulted in a final compendium of 1,427 microarray data sets each surveying transcriptional changes resulting from a different biological context (Table S1).

Expression screening accepts as input this compendium of microarray data as well as a given a query gene set. It then examines each data set in the compendium and calculates for each gene, *g*, the expression correlation between *g* and all other genes. The method uses these correlations to produce a rank ordered list of *g's* expression neighbors and assesses whether the query gene set is significantly over-represented near the top or bottom of this list using an enrichment statistic (see Materials and Methods). This enrichment statistic, following correction for multiple hypothesis testing, serves as a co-expression metric between each gene and the query gene set in that dataset. The procedure is repeated for all datasets in the compendium to generate a co-expression matrix whose values represent each gene's co-expression to the query gene set within a dataset (Figure 1).

Genes that consistently co-express with the query gene set in many independent microarrray datasets likely have a functional role in the query pathway. We therefore sought to generate a measure of consistent coexpression by integrating the co-expression scores for each gene across all data sets. A key feature of the integration scheme is that it offers a principled means of weighting the evidence from each of the data sets. Since the query gene set may itself not be co-expressed in all data sets, we weight data sets according to the intra-correlation of the query gene set to ensure that experiments where the query pathway is itself regulated have greater influence upon the final result. Finally, we apply a data integration procedure that incorporates these weights to arrive at an integrated probability for each gene summarizing its overall co-expression with the query gene set in the microarray compendium (see Materials and Methods). Our data integration procedure is based on the naïve Bayes scheme, which allows independent co-expression evidence from different data sets to strengthen each other, but is modified to be robust against outliers [25],[26]. Importantly, this procedure avoids direct comparison between expression signals from separate data sets, which can introduce artifacts and distort co-expression measures [27],[28].

To validate the expression screening methodology, we first applied it to the well-studied and transcriptionally-regulated cholesterol biosynthesis pathway [29]. We manually curated a set of 19 genes encoding established cholesterol biosynthesis enzymes (Table S2) and applied expression screening to this set. We were able to reconstruct the entire cholesterol biosynthesis pathway within the top 41 high-scoring genes, a substantial improvement over co-expression scores obtained from the best microarray experiment alone (Figure 1B). Among the top 41 co-expressed genes we also recovered the LDL receptor, *SREBF2* and *INSIG1*, three well-known regulators of this pathway (Table S3). A key feature of expression screening is the weighting of each data set according to the intra-correlation of the input pathway. In the case of cholesterol biosynthesis, a variety of data sets representing many distinct biological conditions were given high weights, consistent with the pathway's central role in cellular metabolism (Table S1). Performing data integration without these weights resulted in a substantial loss of specificity (Figure S1). Thus, expression screening is capable of identifying informative datasets in a microarray compendium and reconstructing transcriptionally co-regulated pathways with high precision.

### Application of expression screening to the OxPhos system

**Figure 2:**
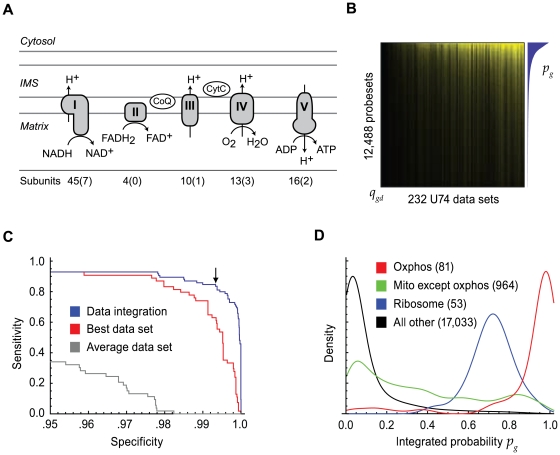
The OxPhos expression screen. (A) A schematic overview of mitochondrial OxPhos. The total number of protein subunits comprising each complex is noted below the schematic. The number of subunits encoded by mitochondrial DNA that were excluded in the OxPhos expression screen is indicated in parentheses. (B) Co-expression matrix from the OxPhos expression screen for the mouse MG-U74Av2 chip. Each value in the matrix represents a gene's (rows) co-expression with OxPhos within a microarray data set (columns). (C) Cross-validated receiver-operator curve reporting the recovery of the query OxPhos genes. Blue line: full expression screen data integration (1,427 data sets). Arrow marks the 99.4% specificity threshold recovering 85% of the OxPhos query genes. Red line: the single best data set. Gray line: a data set with median weight. (D) Histogram of integrated co-expression probabilities from the OxPhos expression screen. Red line: OxPhos query gene set. Green line: non-oxphos mitochondrial genes. Blue line: cytosolic ribosome genes. Black line: all other genes.

We next applied expression screening to the OxPhos pathway using the 1427 microarray dataset compendium, and a manually curated gene set of nuclear-encoded structural OxPhos subunits (Figure 2A, Table S4). We excluded the mtDNA-encoded subunits from the query set since these were not well measured by the Affymetrix platforms. The resulting co-expression matrix (Figure 2B) reveals the robust coordination of OxPhos gene expression in a large variety of biological contexts. The OxPhos gene set exhibits robust intra-correlation (weight *w_d_*>0.75) in nearly 10% of microarray datasets present in the compendium (Table S1). The data set weights enable us to spotlight biological contexts in the compendium for which the modulation of OxPhos gene expression may play an important role. Experiments with large weights include expected conditions such as exercise (GSE1659), Alzheimer's disease (GSE5281) and Pgc1α over-expression (GSE4330) as well as lesser-studied contexts including down-regulation of OxPhos followed by recovery during time-courses of skeletal muscle regeneration (GSE469, GSE5413).

We applied the data integration procedure to identify genes that are consistently co-expressed with OxPhos in the microarray compendium (Table S5). As with the cholesterol biosynthesis pathway, data integration better predicts known genes involved in the OxPhos pathway when compared to the most predictive data set alone (Figure 2C). At a specificity of 99.4%, we were able to recover 85% of the OxPhos pathway (Figure 2C). The integration procedure also lessens confounding correlations with functionally distinct pathways. For example, OxPhos is frequently co-expressed with other genes encoding mitochondrial proteins during mitochondrial biogenesis and turnover, regardless of their specific role in oxidative phosphorylation [30]–[32]. Additionally, OxPhos gene expression may correlate with the expression of other functionally distinct “house-keeping” pathways, especially the cytosolic ribosome, since genes involved in both pathways share a similar set of conserved promoter elements and are controlled by an over-lapping set of transcriptional regulators [33],[34]. In agreement with these findings, we observed significant co-expression (median integrated probability *p_g_* = 0.70) of the cytosolic ribosome with the OxPhos subunits (Figure 2D). However, integrating co-expression across all data sets in the microarray compendium clearly distinguished the OxPhos pathway from other mitochondrial genes and components of the cytosolic ribosome, demonstrating the specificity of expression screening (Figure 2D).

**Figure 3:**
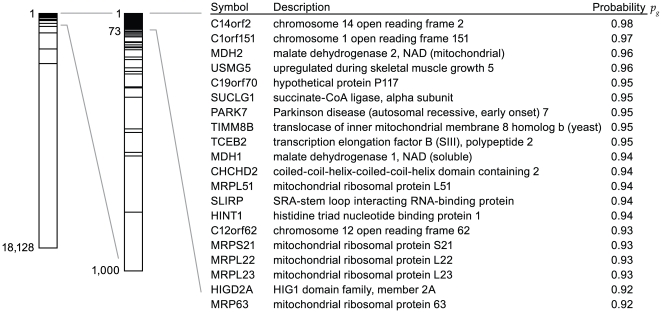
Top scoring genes in the OxPhos expression screen. Left, bar plot indicating the ranks of each OxPhos query gene by descending probability *p_g_*, as well as a magnified view of the 1,000 highest-ranking genes. The table displays the top 20 non-OxPhos genes resulting from the expression screen (corresponding to the top 73 genes including OxPhos).

We next examined the non-OxPhos genes exhibiting the highest co-expression scores. To ensure that co-expression is conserved among mammals, we required that a gene is co-expressed with OxPhos when analyzing human and mouse microarray datasets independently (*p_g_*>0.70 in both species). The top 20 non-OxPhos genes meeting this criterion are shown in Figure 3. Several of the non-OxPhos genes listed in Figure 3 have known metabolic roles in oxidative metabolism such as genes encoding Kreb's cycle enzymes, *MDH2* and *SUCLG1*, as well as several mitochondrial ribosomal subunits necessary for translation of the OxPhos subunits encoded by mtDNA. Other high-scoring genes have never been functionally associated with OxPhos and most lack orthologues in *S. cerevisiae*. It is notable that recent mass spectrometry studies of highly purified mammalian mitochondria have localized every protein present in Figure 3 to the mitochondria with the exception of HINT1, TCEB2 and MDH1, which are primarily cytosolic proteins [35]–[37].

Recently, two candidates identified by our expression screen, C14orf2 and USMG5, have been co-purified with complex V, having been previously missed in purifications of OxPhos due to their small size and biochemical properties (∼7 kD) [38],[39]. While the functions of these two proteins are still unknown, their physical association with complex V further supports the specificity of the expression screening results for identifying OxPhos-related genes. Interestingly, two other uncharacterized proteins presented in Figure 3, C1orf151 and C12orf62, are also less than 10 kD in size (8.6 kD and 6.4 kD, respectively) and contain a single-pass transmembrane domain similar to C14orf2 and USMG5. These molecular similarities suggest that C1orf151 and C12orf62 may also physically associate with OxPhos.

### CHCHD2 and SLIRP are necessary for OxPhos function

To validate the results from the OxPhos expression screen, we selected five functionally uncharacterized mitochondrial candidates from Figure 3 for which we could obtain reliable shRNA reagents to experimentally test their role in OxPhos function (*C14orf2*, *USMG5*, *CHCHD2*, *SLIRP* and *PARK7*). For each of the five candidate genes, we identified at least two independent, non-toxic shRNAs that deplete mRNA abundance by more than 85% (Figure S2). We were unable to obtain high quality shRNA reagents for other candidates including *C1orf151*, *C19orf20* and *C12orf62*.

**Figure 4:**
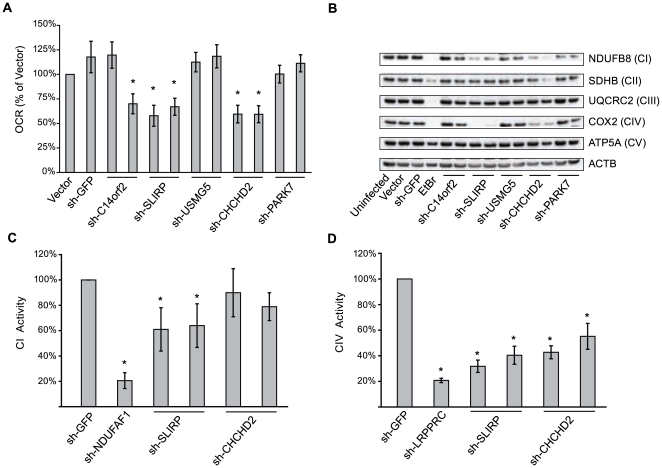
Silencing *CHCHD2* and *SLIRP* disrupts OxPhos function. (A) Oxygen consumption rate (OCR) of MCH58 human fibroblasts measured at 10–14 days post-infection with empty vector, shRNA targeting GFP, or two independent shRNAs targeting each of five candidates from the OxPhos expression screen. Values are reported as percent of empty vector sample's OCR and represent means of 20 replicate wells. Error bars indicate the 95% normal confidence interval. (B) Western blot of cleared whole cell lysate harvested 10–14 days post-infection, subjected to SDS-Page and blotted for labile markers of each OxPhos complex. shRNA targets are indicated below each lane and blotted proteins are indicated to the right with their respective OxPhos complex in parentheses. EtBr represents a positive control sample from cells treated with 40 ng/ml ethidium bromide for 4 days. (C, D) Assays for activity of complexes I and IV in whole-cell native protein harvested from MCH58 cells infected in triplicate with shRNAs targeting *SLIRP*, *CHCHD2* (two independent shRNAs each) or GFP. Values are reported as percent of activity for shRNA targeting GFP. Error bars represent standard deviation. *, P<0.05 (n = 3, two-tailed unpaired t-test).

We first silenced each candidate gene in immortalized human fibroblasts and measured the live-cell oxygen consumption rate (OCR) as a general parameter of basal OxPhos activity. Silencing of two candidates, *CHCHD2* and *SLIRP*, significantly reduced cellular OCR by approximately 40% compared to control cells (P<.05; Figure 4A). Additionally, a single shRNA targeting *C14orf2* reduced OCR by 35% (P<.05); however, this result may be due to an off-target effect since a second hairpin targeting *C14orf2* did not substantially affect OCR (Figure 4A).

Inherited or acquired mutations causing OxPhos dysfunction often destabilize or cause the misassembly of one or more of the five complexes comprising OxPhos. We therefore assessed whether knock-down of any of the five candidates affected complex stability by blotting for “labile” OxPhos subunits whose stability depends on their respective complex being properly assembled in the mitochondrial inner membrane (Figure 4B). Again, we noted that knock-down of *SLIRP* and *CHCHD2* clearly affected OxPhos as both dramatically reduced the abundance of the complex IV subunit, COX2, and to a lesser extent, NDUFB8, a component of complex I. To ensure that *CHCHD2* and *SLIRP* are responsible for maintaining the activities of OxPhos complexes I and IV in native form, we measured the activity of immuno-captured preparations of these complexes (Figure 4C and 4D) [40]. Reducing the expression of both candidates reduced cellular CIV activity (P<.05) while only *SLIRP* significantly affected CI (P<.05).

### SLIRP is an RNA–binding protein that maintains mitochondrial RNA expression

The SLIRP protein contains an RNA-binding domain and was previously reported to associate with steroid receptor RNA activator (SRA), a nuclear non-coding RNA, and thereby repress the ability of SRA to activate nuclear receptors [41]. However, SLIRP is predominantly mitochondrial [35],[41]. Since the protein is localized to the mitochondria and is able to bind RNA, we hypothesized that it might affect OxPhos activity by directly modulating the level of mtRNA, either through expression, processing or stability of the mitochondrial transcripts. mtRNA is transcribed from mtDNA in two continuous poly-cistronic transcripts (one from each mtDNA strand), which are subsequently processed to produce eleven OxPhos protein-encoding mRNAs, two ribosomal RNAs (rRNA) and a full complement of tRNAs. The processed mtRNAs are individually regulated by mtRNA stability factors, many of which remain to be identified [42].

**Figure 5:**
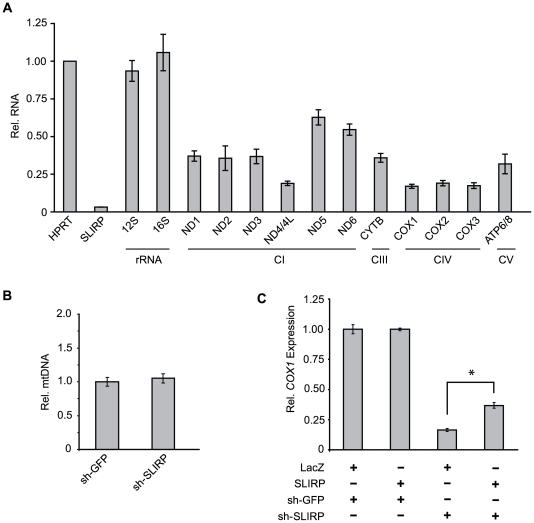
SLIRP maintains mitochondrial mRNA. (A) Expression levels of mitochondrial transcripts in MCH58 cells measured by qPCR 10 days post-infection with either control shRNA (shGFP) or shRNA targeting *SLIRP*. Values are reported as fold change over shGFP-treated cells, in each case normalized to *HPRT* as an endogenous control, and represent means (n = 3). All values except *16S* and *12S* were significantly decreased compared to shGFP (P<.05, two-tailed unpaired t-test). (B) mtDNA copy-number per cell measured by qPCR using genomic DNA from the samples in (A). Values represent mean mtDNA/nuclear DNA ratio (n = 3). (C) *COX1* expression measured by qPCR in MCH58 cells infected with shSLIRP or shGFP. Labels LacZ and SLIRP indicate cells transfected with constructs to over-express LacZ or human SLIRP, respectively. Values reported are mean ratios over LacZ+shGFP (n = 3). Error bars indicate standard deviation. *, P<.05 (two-tailed unpaired t-test).

To determine whether SLIRP acts in the mtRNA processing pathway, we designed a full panel of qPCR assays to measure the abundance of each protein-coding and ribosomal mtRNA transcript (Table S5). We again used shRNA to reduce *SLIRP* expression and measured the resulting effect on each mtRNA transcript. Knock-down of *SLIRP* significantly reduced the abundance of all eleven protein-encoding mtRNA transcripts (Figure 5A), while the mtDNA copy-number was unaffected (Figure 5B). The most pronounced mtRNA reduction was seen for transcripts encoding complex IV subunits as well as the bi-cistronic transcript encoding the ND4 and ND4L subunits of complex I, which is concordant with the specific complex I and IV biochemical defects shown in Figure 4. The effect of SLIRP depletion upon mtRNA appears specific to the protein-encoding mtRNA transcripts since it did not affect the expression of the *12S* or *16S* mitochondrial rRNAs (Figure 5A), even though these rRNAs are encoded on the same primary poly-cistronic transcript that contains all but one of the mitochondrial mRNAs. To assess whether this regulation of mtRNA by SLIRP is conserved among mammals, we also silenced the gene encoding the mouse ortholog of SLIRP in C2C12 myoblasts. We again observed down-regulation of all three complex IV-encoding mtRNAs (Figure S3).

Since SLIRP is proposed to be alternatively localized to the nucleus, we wondered whether it might affect mtRNA expression indirectly by regulating the nuclear expression of known mtDNA transcription factors or mtRNA regulators. However, shRNA targeting *SLIRP* did not significantly alter the expression of known nuclear-encoded mtRNA regulators *TFAM*, *TFB1M*, and *TFB2M*, nor did it affect the expression of the nuclear-encoded OxPhos subunit *UQCRC1*, further suggesting that SLIRP acts within the mitochondria to regulate mtRNA abundance (Figure S4). Finally, we investigated whether over-expression of SLIRP would be sufficient to boost mtRNA abundance in the cell. Over-expressing SLIRP for 48 hours did not alter mtRNA abundance, but over-expression did rescue the down-regulation of mtRNA resulting from knock-down of SLIRP in human cells. Besides demonstrating that the over-expression construct is functional and that the shRNA is on-target, this indicates that SLIRP is not a limiting factor for mtRNA abundance in wild-type cells (Figure 5C).

### The SLIRP protein depends upon mtRNA for its own stability

**Figure 6:**
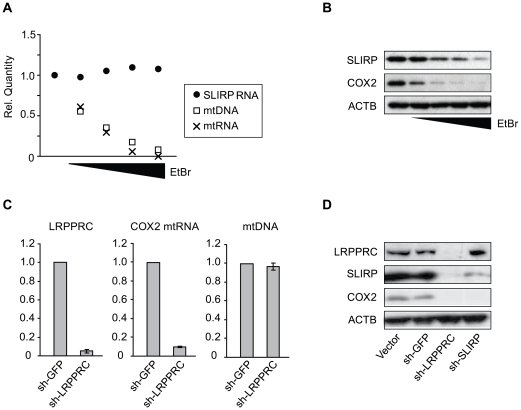
SLIRP depends upon mitochondrial mRNA for stability. (A) mtDNA copy-number, *mtND2* expression (mtRNA), and *SLIRP* expression measured by qPCR from MCH58 cells depleted of mtDNA for 4 days in the presence of 2.5, 5, 10, or 20 ng/ml ethidium bromide. (B) Western blot of SLIRP, COX2, and ACTB protein abundance for the samples in (A). (C) mtDNA copy-number and mRNA expression of COX2 and LRPPRC in MCH58 cells infected with shRNA targeting LRPPRC or a GFP control. Mean ratios over shGFP cells are reported (n = 3). (D) Western blot of protein lysates from shGFP-, shSLIRP-, and shLRPPRC-infected cells 10 days post-infection.

Since adequate expression of *SLIRP* is essential for maintaining mtRNA levels, we asked whether *SLIRP* is transcriptionally regulated in response to a depletion of mtRNA. We used ethidium bromide (EtBr), a DNA-intercalating agent that is selectively absorbed by mitochondria and reduces mtDNA copy-number and mtRNA expression in the cell [43]. Following treatment with EtBr for four days, we did not observe any compensatory increase in *SLIRP* expression as mtDNA and mtRNA were depleted in a concentration-dependent manner (Figure 6A). Surprisingly, however, we did observe a dramatic reduction in SLIRP at the protein level, in a manner depending on the concentration of EtBr (Figure 6B). This suggests that the stability of SLIRP depends upon either mtDNA copy-number or mtRNA abundance. A similar phenomenon has been previously reported for TFAM, a critical regulator of both mitochondrial DNA and RNA [30]. TFAM coats the mtDNA to protect it from degradation but TFAM is also dependent upon mtDNA for its own protein stability [44]. We wondered whether SLIRP, being an RNA-binding protein, depends exclusively upon mtRNA for its stability. To assess whether mtRNA rather than mtDNA quantity is important for stabilizing SLIRP we used shRNA to deplete cells of LRPPRC, a mitochondrial protein necessary for maintaining mtRNA expression but not mtDNA copy-number [45] (Figure 6C). We again observed a substantial drop in SLIRP protein abundance, likely indicating a mutual partnership between SLIRP and mtRNA where each is responsible for the other's stability within the mitochondria (Figure 6D).

## Discussion

To predict novel OxPhos regulators we have developed a method called expression screening, which utilizes the inherent strong transcriptional co-expression of the known OxPhos structural subunits to identify other genes sharing similar expression profiles in a compendium of microarray data. While co-expression analysis alone cannot fully distinguish functionally relevant co-regulation from mere correlation, we have demonstrated that the integration of evidence from hundreds of biological contexts significantly enhances predictive power. In this manner, we were able to build a reliable classifier for membership in the OxPhos pathway that will be a useful resource for prioritizing candidate genes in patients with respiratory chain disorders.

Expression screening predicted several functionally uncharacterized genes as novel regulators of the OxPhos pathway. Of these, *CHCHD2* and *SLIRP* resulted in clear OxPhos deficits when targeted by shRNA. CHCHD2 is a member of a family of proteins containing the CHCH domain (coiled-coil-helix-coiled-coil-helix; PFAM#06747). Conserved cysteines within this motif have previously been implicated in metal coordination or transport suggesting that CHCHD2's role in stabilizing complex IV may be related to regulation of the complex IV copper centers [46]. Interestingly, the CHCH domain is also found in an OxPhos complex I subunit, NDUFA8, and in another Complex IV assembly factor, COX19 [46],[47]. Further study of this important domain should lend insight to the assembly and function of OxPhos.

Silencing of some high-scoring expression screening candidates including *USMG5*, *C14orf2* and *PARK7* did not result in an oxidative phenotype. These outcomes may reflect common caveats with shRNA experiments including insufficient protein knock-down, functional redundancy or lack of the proper experimental context. For example, our expression screen implicates the gene *PARK7*, named for causing Parkinson's disease when mutated, as a key player in OxPhos biology. Currently, there is no established role for PARK7 in the OxPhos pathway [48]. While we did not observe an effect on basal oxygen consumption when perturbing *PARK7* expression in our cell line, PARK7 protein may still be an important OxPhos regulator that acts in a context-dependent manner. Others have reported PARK7 to act as an antioxidant that scavenges mitochondrial radical oxygen species, a harmful by-product of an active OxPhos system [49]. Additionally, cells depleted of PARK7 are hyper-sensitive to rotenone treatment, a potent complex I inhibitor [50].

*SLIRP* is consistently co-expressed with the nuclear OxPhos machinery and regulates the abundance of the mitochondrial protein-encoding transcripts. These properties raise the interesting possibility that *SLIRP* is co-regulated with the nuclear OxPhos genes in order to coordinate nuclear and mitochondrial OxPhos gene expression. This phenomenon has also been previously reported for genes encoding the mtDNA transcription factors: *TFAM*, *TFB1M* and *TFB2M* [51],[52]. In certain biological contexts, these genes have been noted to be co-expressed with nuclear OxPhos genes [51],[52]. In our expression screen, we did observe co-expression of these factors with OxPhos in certain microarray experiments, but this co-expression was not frequent enough to generate a high score in the overall data integration. In contrast, *SLIRP* scored among the top 20 genes in the genome for its co-expression with OxPhos (Figure 3), strongly implicating a role for *SLIRP* in synchronizing nuclear and mitochondrial gene expression.

The precise molecular mechanism by which SLIRP maintains mtRNA is not yet clear. To date, most studies of mtRNA maintenance has focused upon the core transcriptional machinery responsible for transcribing the primary poly-cistronic transcripts. This essential machinery includes the mitochondrial polymerase, POLRMT and its partner MRPL12, as well as the transcription factors TFAM, TFB1M and TFB2M [3]. However, key factors in mammalian mtRNA post-transcriptional processing and stability remain unknown [24]. For example, a human mitochondrial poly(A) polymerase (mtPAP) has been recently identified [53], but this protein does not contain an obvious RNA-binding domain, suggesting that it requires one or more currently unidentified RNA-binding partners [24]. Additionally, in *S. cerevisiae*, several mitochondrial RNA-binding proteins stabilize mtRNA transcripts, but proteins with similar functions have not been found in mammals [54]–[56]. Given its involvement in maintaining mtRNA it is tempting to speculate that SLIRP fulfills one or more of these roles in mammalian mtRNA biology. SLIRP joins a small cast of RNA-binding mitochondrial proteins that are responsible for maintaining steady-state mtRNA in human cells: LRPPRC, SUPV3L1 and PTCD2 [45],[57],[58]. LRPPRC is of central importance for OxPhos function, and patients harboring mutations in *LRPPRC* develop the French-Canadian variant of Leigh syndrome, a devastating hepatocerebral metabolic disorder [11]. In our study, shRNA targeting *SLIRP* phenocopies mutations in *LRPPRC* by causing loss of mtRNA and a significant reduction in complex IV activity. Given these similarities, *SLIRP* should be considered a candidate gene for respiratory chain disorders.

Many of the proteins encoded by the human genome are still functionally uncharacterized and methods such as expression screening will be useful in closing the gaps in our knowledge of cellular pathways [59]. While we have applied expression screening to the cholesterol biosynthesis and OxPhos pathways, this technique is readily extendible to any gene set exhibiting transcriptional co-regulation. In a separate study we show the utility of this method in identifying new mitochondrial proteins important for heme biosynthesis [60]. Expression screening is not the only tool that should be considered for functional prediction. Network-based integration of protein-protein interaction data and other data integration methods have also been successfully applied to predict the functions of uncharacterized proteins [19],[61],[62]. Combining expression screening with these or other methods could possibly yield more accurate predictions, especially in cases where transcriptional regulation may not be the dominant mode of regulatory control. Still, microarray gene expression data has several advantages: it is by far the most abundant data source available, offers a more unbiased approach than most techniques, and permits investigating gene function in specific biological contexts. As public repositories of microarray data continue to grow at an accelerating pace, we anticipate that expression screening will become an increasingly important tool for discovering gene function.

## Materials and Methods

### Expression screening

A total of 2,052 mouse and human microarray data sets were downloaded from the NCBI Gene Expression Omnibus [18] in March 2008 for the Affymetrix platforms HG-U133A (GEO GPL96), HG-U133+ (GEO GPL570), MG-U75Av2 (GEO GPL81), Mouse430A (GEO GPL339) and Mouse430 (GEO GPL1261). We discarded data sets with less than 6 arrays as well as data sets containing multiple tissues. We then merged overlapping data so that no two data sets shared identical arrays, resulting in a final compendium of *D* = 1,427 data sets. For a given query gene set *S*, each data set *d* and each gene *g*, we calculated the vector of Pearson correlation coefficients *r_gj_* between *g* and all other genes *j*. We then define the correlation between *g* and *S* as the GSEA-P enrichment score (ES) statistic [63] with *g* as the “phenotype” variable and N representing the total number of genes.
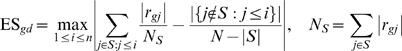



We next calculated randomized enrichment scores ES^0^ by randomly permuting values (arrays) for gene *g*, re-calculating *r_gj_* and applying the above formula. We pooled *N*_0_ = 100,000 permuted ES^0^ values from all *N* genes to estimate the marginal null distribution of enrichment scores. From this we estimated the global false discovery rate (FDR) of each actual ES value [63],[64] as the ratio of tail probabilities:
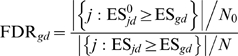



We take *q_gd_* = 1−FDR*_gd_* to represent the probability of co-expression of gene g with the query gene set *S* in data set d. The data set weights *w_d_* were defined as the average of *q_gd_* across the query genes. We then integrated these probabilities using a robust Bayesian formula [25] to obtain a final probability *p_g_* of co-expression of gene g with the query gene set,
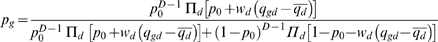
where 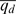 is the average of *q_gd_* in data set *d*. This method of data integration assumes conditional independence between data sets given the co-expression hypothesis, which allows concurring evidence from multiple data sets to reinforce each other in calculating the integrated probability. Incorporating the prior *p*_0_ affords some robustness to outliers in terms of *q_gd_* values close to 0 or 1, which can arise from the permutation-based FDR estimation. For the OxPhos expression screen the prior *p*_0_ was set to 5%, roughly corresponding to the fraction of mitochondrial genes in the genome [35]. Since the query genes are used to calculate the weights *w_d_*, the sensitivity and specificity was estimated using leave-one-out cross-validation, with one gene withheld from the weights calculation in each iteration.

### Mapping genes to Affymetrix probesets

We mapped Affymetrix probesets to NCBI Homologene identifiers using a previously described method [65]. For the query gene sets (OxPhos and cholesterol pathways) we validated each gene's mapping by Blast. It is important that each query gene is represented only once in the gene set. For cases in which multiple Affymetrix probesets map to a gene in the query gene set, we chose the probeset with the least potential for cross-hybridization according to Affymetrix probeset annotations. Specifically, we used the following Affymetrix probeset suffix hierarchy (‘at’>‘a_at’>‘s_at’>‘x_at’). In cases where there were ties, we chose the lower numbered probeset to represent a gene.

We performed expression screening as described above separately for each microarray platform, using Affymetrix probeset-level data. When integrating across all array platforms, we chose for each Homologene identifier the probeset with maximal *p_g_* in the platform-specific screen. We then re-integrated across all data sets, ignoring missing values, to produce the final list of probabilities, *p_g_* (Figure 3, Table S3, Table S5).

### Cell culture and media

MCH58 immortalized human fibroblasts were kindly donated by Eric Shoubridge [66]. 293T and C2C12 cells were received from the American Type Culture Collection (CRL1772 & CRL11268). Unless otherwise indicated, all experiments were carried out in DMEM, 4.5 g/L glucose, 10% FBS (Sigma #2442) supplemented with 2 mM glutamine, 100 I.U Penicillin and 100 ug/ml Streptomycin.

### shRNA lentivirus production and infection

Lentiviral vectors for expressing shRNA (pLKO.1) were received from the Broad Institute's RNAi Consortium or from Open Biosystems. Unique identifiers of each shRNA construct can be found in Figure S1. Procedures and reagents for virus production are adapted from the Broad Institute's RNAi Consortium protocols [67]. Briefly, 400,000 293T cells were seeded in a 24-well dish and 12 hr later triple-transfected with pLKO.1-shRNA, a packaging plasmid (pCMV-d8.91) and a pseudotyping plasmid (pMD2-VSVg) using Fugene6 reagent at 3∶1 (reagent∶DNA) (Roche #11815091001). Media was refreshed 18 hr post-transfection, supplemented with 1% BSA and virus collected 24 h later. For infection, 30,000 cells were seeded in a 24-well dish. 30 ul viral supernatant was added to cells for a final volume of 500 ul media containing 8 ug/ml polybrene (Sigma #H9268). The plates were spun 800rcf for 30 min at 32°C, returned to 37°C and 24 h post-infection were selected for infection with 2 ug/ml puromycin (Sigma #P9620). RNA for assessing knock-down efficiency was isolated 7–10 days post-infection.

### cDNA and qPCR

RNA was isolated using the RNeasy system (Qiagen #74106) with two repetitions of DNAse digestion to remove mtDNA and genomic DNA from the sample. 1 ug of RNA was used for 1^st^-strand cDNA synthesis using a mix of poly(dT) and random hexamer primers (SuperScript III, Invitrogen, #18080). Genomic DNA used for the analysis of mtDNA quantity per cell was isolated using Qiagen DNeasy system. 1 ng genomic DNA was used for multiplex qPCR analysis to simultaneously measure nuclear DNA and mtDNA (See Table S6 and [68]). qPCR of cDNA and genomic DNA was performed using the 96-well ABI7500 qPCR system in 20 ul reactions prepared with 2× master-mix (ABI #4369510), the appropriate 20× ABI taqman assay (Table S6) and diluted cDNA sample.

### SLIRP over-expression

A pDONR-221 Gateway clone for *SLIRP*, used previously by our laboratory for cellular localization studies [35], was sequence verified and cloned into a pcDNA destination vector in-frame with a C-terminal V5-His tag (pDEST40, Invitrogen #12274-015). 293T cells were infected with validated shRNAs targeting either *SLIRP* or *GFP* as described above. Seven days post-infection, 400,000 cells were seeded in a 24-well dish and transfected with either *pcDNA-LacZ-V5-His* or *pcDNA-SLIRP-V5-His*. 48 hrs post-transfection, cells were harvested for downstream analyses.

### Live-cell oxygen consumption measurements

Live-cell oxygen consumption readings (OCR) were performed using a 24-well Seahorse XF24 Bioflux analyzer. MCH58 cells were seeded at a density of 30,000/well in unbuffered media (4.5 g/L glucose, 4 mM glutamine, 1 mM pyruvate in DMEM, pH 7.4). The XF24 analyzer was set to read OCR/well as an average of four 3 min measurements with a 1 min mixing interval between measurements. Each plate contained four different samples of five replicates each with one sample always being the shRNA vector control (pLKO.1) that was used for normalization and comparison between experiments. Each “batch” of four samples was measured on four different plates summing to 20 replicates per sample. Samples were randomized within each plate to avoid plate-position effects and experiments were repeated multiple times to ensure reproducibility. To correct for cell seeding errors, we measured the total protein content per well after each experiment (BCA method) and normalized the OCR per well by dividing by the corresponding protein concentration.

### Immunoblotting

5 ug of cleared whole cell lysate isolated in RIPA buffer was used per lane on a 4–12% Bis-Tris gel (Invitrogen, #NP0321) and blotted on a PVDF membrane (Invitrogen, #LC2005) using a semi-dry transfer apparatus (Bio-Rad), 15 V, 20 min. Membranes were blocked for 2 hr at room temperature in tris-buffered-saline solution (Boston BioProducts #BM300) with .1% Tween-20 and 5% BSA (TBS-T-BSA). Primary antibodies were incubated with the membrane overnight at 4°C in TBS-T-BSA at the dilutions reported in Table S7. Secondary sheep-anti-mouse (GE-HealthCare, #na931v) or sheep-anti-rabbit (GE-HealthCare, #na934v) antibodies were incubated with the membrane at a 1∶5,000 dilution in TBS-T-BSA for 45 min at room temperature. The membrane was developed using Super Signal West Pico (Pierce, #34077).

### OxPhos Complex I and IV activity measurements

Complex I and Complex IV Dipstick activity assays were performed on 20 ug and 25 ug cleared whole cell lysate, respectively, according to the manufacturer's protocol (Mitosciences #MS130 and #MS430). 30 ul of lysis buffer A was used per 500,000 cells for lysis and solubilization.

## Supporting Information

Figure S1 Validation of data set weighting. ROC curve comparing the original formulation of expression screening for the cholesterol gene set (blue line) versus a screen using uniform weights (red line).
(0.34 MB PDF)

Figure S2 Validation of gene silencing by RNAi. mRNA expression in MCH58 human fibroblasts infected with lentivirus to over-express shRNAs targeting each candidate gene (see Materials and Methods). RNA was isolated 5–7 days post-infection and used to produce cDNA for measuring RNAi efficiency by qPCR (see Materials and Methods). Each column represents an independent shRNA hairpin targeting the indicated gene. Hairpins are labeled by their official Broad Institute RNAi consortium identifiers. Reported values are normalized to RNA from uninfected cells. Error bars represent the range of the duplicate measurements. Arrows indicate the best two shRNAs for each gene which were used for downstream experiments.
(0.56 MB PDF)

Figure S3 Silencing the SLIRP homologue in mouse cells reduces mtRNA expression. (A) mRNA expression levels of C2C12 mouse myoblasts infected with shRNA targeting different regions of *1810035L17Rik*, the mouse homologue of *SLIRP*. RNA was isolated five days post-infection and *1810035L17Rik* mRNA abundance was measured by qPCR. Error bars represent the range of duplicate measurements. (B) mRNA levels for *Actin*, *1810035L17Rik*, *COX1*, *COX2*, and *COX3* in C2C12 mouse myoblasts infected with shRNA TCRN0000103890 targeting *1810035L17Rik*. Values are given as ratios over shGFP-treated cells. mtDNA quantity was measured from genomic DNA isolated simultaneously with RNA. Error bars represent standard deviation (n = 3). *, P<.05 (two-tailed unpaired t-test).
(0.33 MB PDF)

Figure S4 Silencing *SLIRP* does not affect *UQCRC1*, *TFAM*, *TFB1M*, or *TFB2M* expression. mRNA levels of *UQCRC1*, *TFAM*, *TFB1M*, and *TFB2M* measured by qPCR from the samples described in Figure S2. SLIRP_A and SLIRP_B are independent shRNA hairpins corresponding to Figure S2 samples TRCN0000152814 and TRCN0000154330, respectively. Results were normalized to the expression of *HPRT* as an endogenous control. Values are reported as average ratio over shGFP; error bars indicate standard deviation (n = 3).
(0.15 MB PDF)

Table S1 Microarray data sets used in expression screening. Each microarray data set is listed with its GEO accession number, the data set title and Affymetrix platform. Mouse platforms are preceded by “MG” while human platforms are preceded by “HG”. Each data set is also listed with its “weight” representing the intra-correlation of either the OxPhos gene set or cholesterol pathway gene set (see Materials and Methods).
(0.28 MB XLS)

Table S2 The cholesterol pathway gene set used in expression screening. Genes directly involved in cholesterol biosynthesis were mapped to each Affymetrix platform. In cases where multiple probesets match a gene, we chose the probeset exhibiting the least cross-hybridization at the sequence level according to Affymetrix annotations.
(0.02 MB XLS)

Table S3 Results of the cholesterol biosynthesis expression screen. The top 41 genes resulting from the cholesterol expression screen sorted by their probability of co-expression with the cholesterol gene set after data integration of all human and mouse microarray data sets (“Human&Mouse” column). We also include the probabilities resulting from data integration of each platform alone or of each species, human or mouse, alone. The Affymetrix probeset yielding the highest probability of co-expression for each gene is given for each platform. Genes are identified by their NCBI homologene ID (HID). Cholesterol pathway genes used as the query gene set are identified by a “1” in the “Cholesterol” column.
(0.03 MB XLS)

Table S4 The OxPhos pathway query gene set. Genes previously validated as structural subunits of OxPhos (“citation” column) were mapped to Affymetrix platforms (see Materials and Methods). Genes used in the query set of expression screening are marked by an asterisk. Some OxPhos structural subunits are encoded by the mitochondrial genome and are not well measured by Affymetrix platforms. Additionally, some subunits are tissue specific or are only present in either human or mouse. These are shown in the table but were not included in the query gene set.
(0.05 MB XLS)

Table S5 Results of the OxPhos expression screen. For each gene, we report the probability of co-expression with the OxPhos gene set after data integration of all human and mouse microarray data sets (“Human&Mouse” column). We also include the probabilities resulting from data integration of each platform alone or from human or mouse alone. The Affymetrix probeset yielding the highest probability of co-expression for each gene is given for each platform in the rightmost columns. When there is no probeset listed for a gene in a particular platform, that gene is not measured by the platform and the platform is not included in the data integration procedure. Genes are identified by their NCBI homologene ID (HID) and Entrez gene symbol. OxPhos genes used in the query gene set are identified by a “1” in the “OxPhos” column.
(5.74 MB XLS)

Table S6 Taqman qPCR assay information.
(0.02 MB XLS)

Table S7 Antibodies used in this study.
(0.01 MB XLS)
