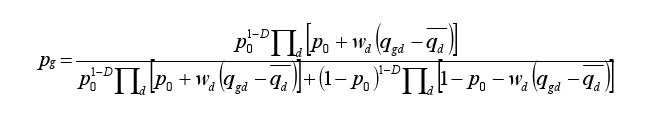# Correction: A Computational Screen for Regulators of Oxidative Phosphorylation Implicates SLIRP in Mitochondrial RNA Homeostasis

**DOI:** 10.1371/annotation/36fe7624-0904-46d4-a013-4be6195245c4

**Published:** 2010-03-01

**Authors:** Joshua M. Baughman, Roland Nilsson, Vishal M. Gohil, Daniel H. Arlow, Zareen Gauhar, Vamsi K. Mootha

In the third equation under Materials and Methods, the exponent term 1-D was incorrectly rendered as D-1. Please view the correct equation here: