# Supplementary figures and images for: A Computational Screen for Regulators of Oxidative Phosphorylation Implicates SLIRP in Mitochondrial RNA Homeostasis
Source: PLoS Genet. 2009 Aug 14;5(8):e1000590. doi: 10.1371/journal.pgen.1000590 (PMC2721412; doi:10.1371/journal.pgen.1000590)

Figure S1

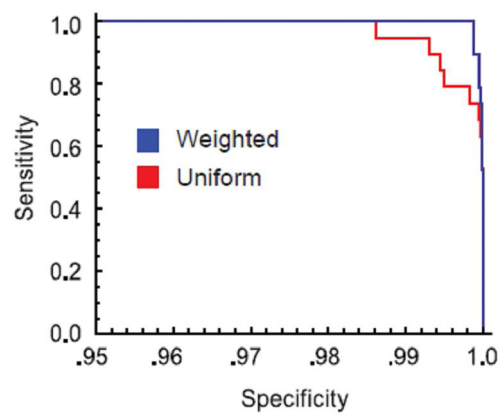

Supplement: Figure S1 — Validation of data set weighting. ROC curve comparing the original formulation of expression screening for the cholesterol gene set (blue line) versus a screen using uniform weights (red line). (0.34 MB PDF) [file pgen.1000590.s001.pdf]

Figure S2

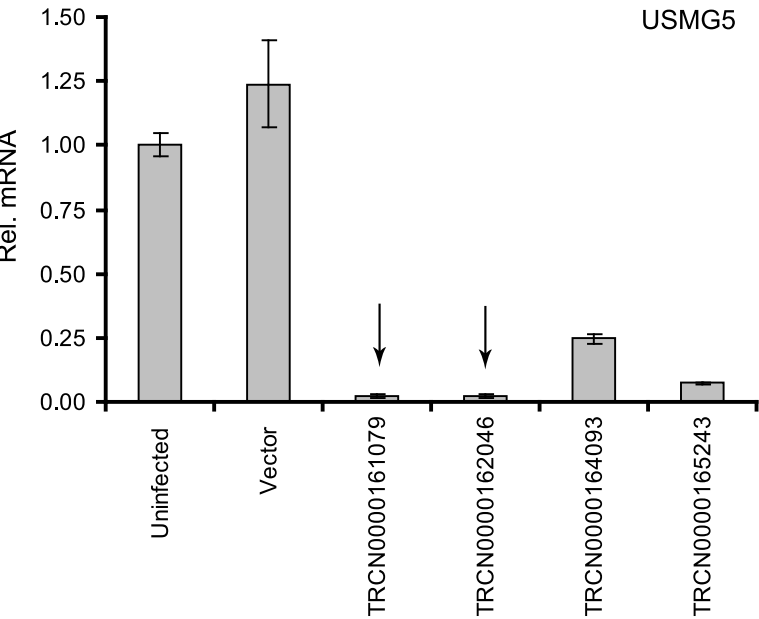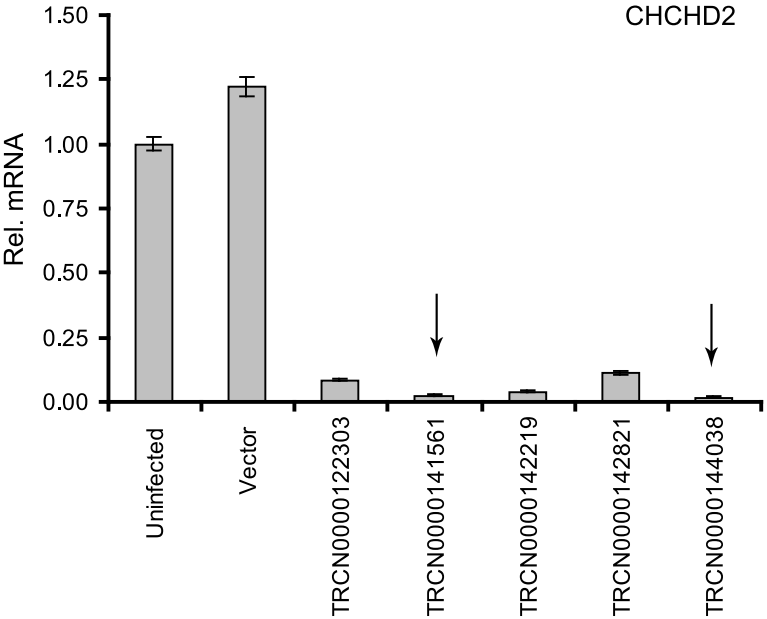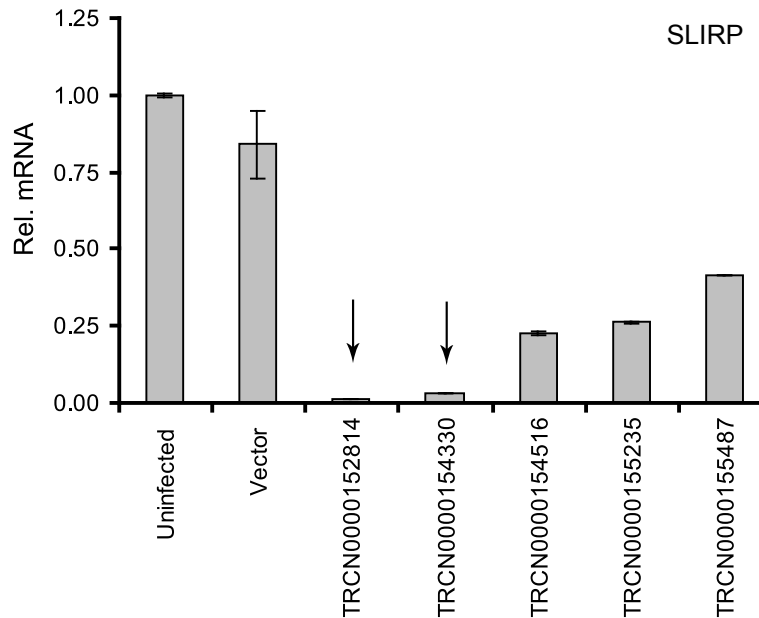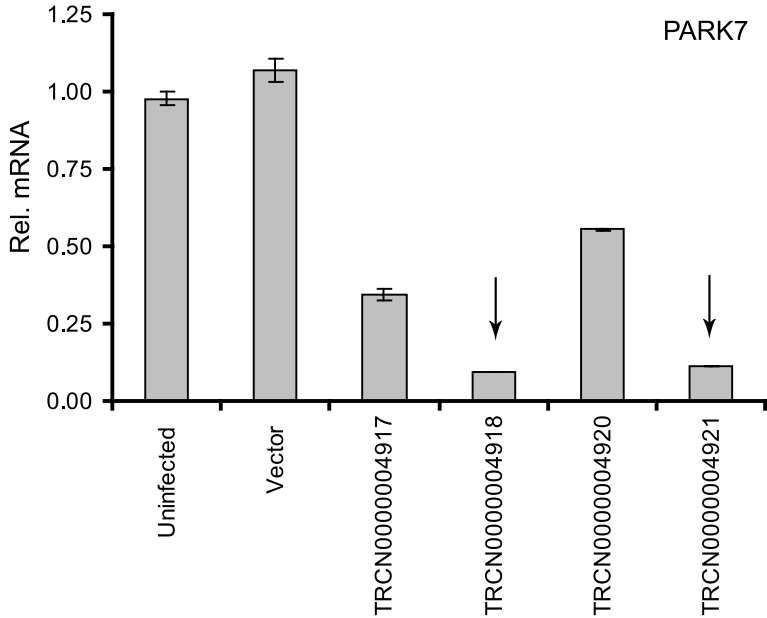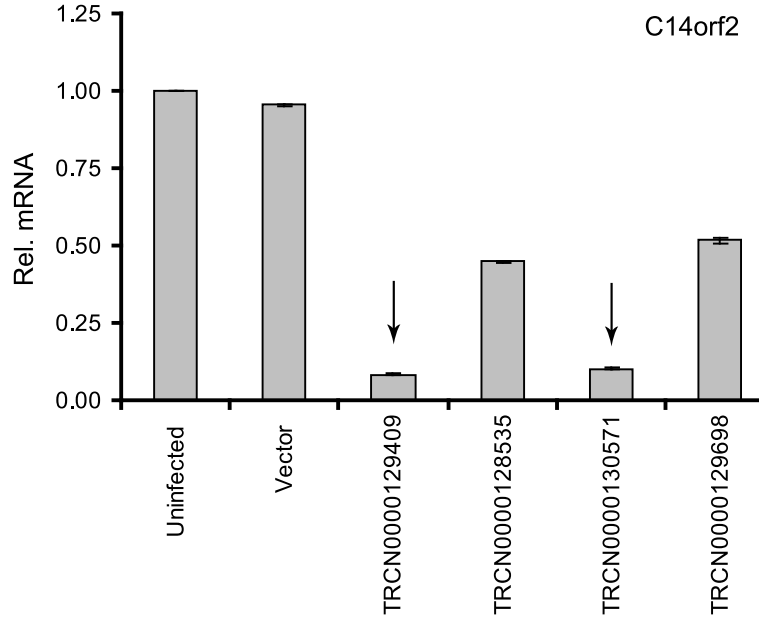

Supplement: Figure S2 — Validation of gene silencing by RNAi. mRNA expression in MCH58 human fibroblasts infected with lentivirus to over-express shRNAs targeting each candidate gene (see Materials and Methods). RNA was isolated 5–7 days post-infection and used to produce cDNA for measuring RNAi efficiency by qPCR (see Materials and Methods). Each column represents an independent shRNA hairpin targeting the indicated gene. Hairpins are labeled by their official Broad Institute RNAi consortium identifiers. Reported values are normalized to RNA from uninfected cells. Error bars represent the range of the duplicate measurements. Arrows indicate the best two shRNAs for each gene which were used for downstream experiments. (0.56 MB PDF) [file pgen.1000590.s002.pdf]

Figure S3

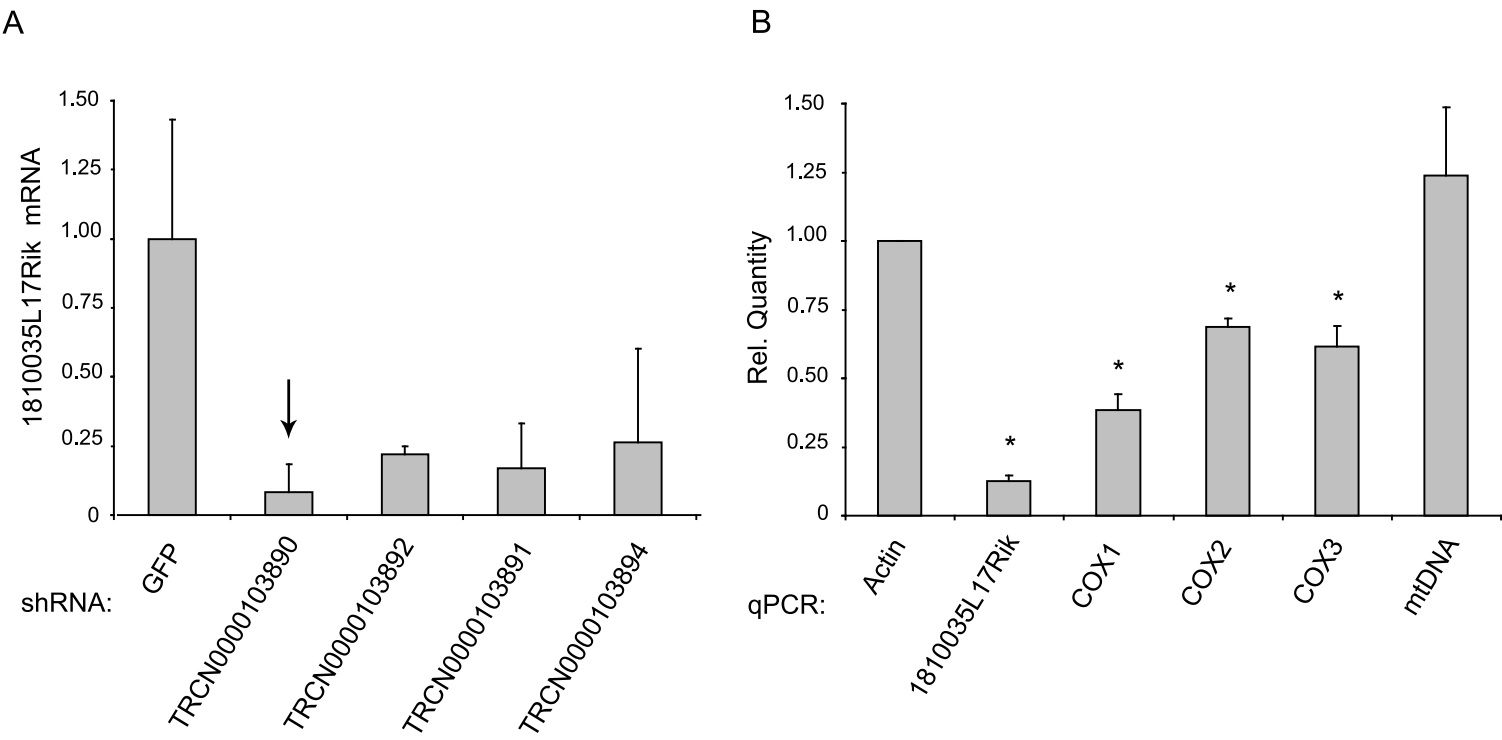

Supplement: Figure S3 — Silencing the SLIRP homologue in mouse cells reduces mtRNA expression. (A) mRNA expression levels of C2C12 mouse myoblasts infected with shRNA targeting different regions of 1810035L17Rik, the mouse homologue of SLIRP. RNA was isolated five days post-infection and 1810035L17Rik mRNA abundance was measured by qPCR. Error bars represent the range of duplicate measurements. (B) mRNA levels for Actin, 1810035L17Rik, COX1, COX2, and COX3 in C2C12 mouse myoblasts infected with shRNA TCRN0000103890 targeting 1810035L17Rik. Values are given as ratios over shGFP-treated cells. mtDNA quantity was measured from genomic DNA isolated simultaneously with RNA. Error bars represent standard deviation (n = 3). *, P<.05 (two-tailed unpaired t-test). (0.33 MB PDF) [file pgen.1000590.s003.pdf]

Figure S4

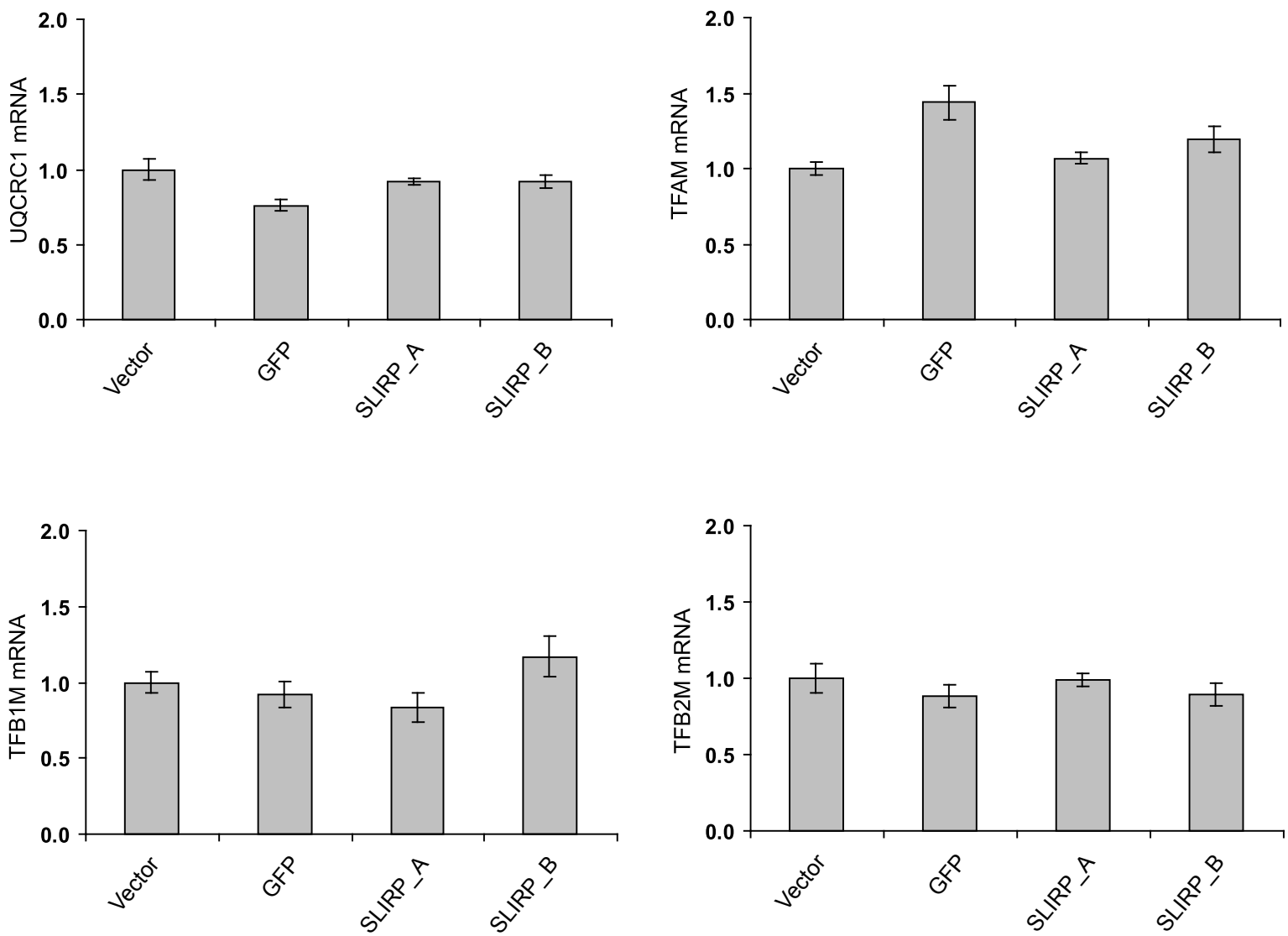

Supplement: Figure S4 — Silencing SLIRP does not affect UQCRC1, TFAM, TFB1M, or TFB2M expression. mRNA levels of UQCRC1, TFAM, TFB1M, and TFB2M measured by qPCR from the samples described in Figure S2. SLIRP_A and SLIRP_B are independent shRNA hairpins corresponding to Figure S2 samples TRCN0000152814 and TRCN0000154330, respectively. Results were normalized to the expression of HPRT as an endogenous control. Values are reported as average ratio over shGFP; error bars indicate standard deviation (n = 3). (0.15 MB PDF) [file pgen.1000590.s004.pdf]
